# Dual-Modal Autofluorescence Microscopy for CycleGAN-Based H&E-like Image Generation in Liver Tissue: A Preliminary Proof-of-Concept Study

**DOI:** 10.3390/diagnostics16131993

**Published:** 2026-06-26

**Authors:** Qiuying Li, Chunyang Fan, Bing Li, Xiaoxu Liu, Zhuo Zhao, Zheng Wang

**Affiliations:** 1 Key Laboratory of Shaanxi Province for Craniofacial Precision Medicine Research, College of Stomatology, Xi’an Jiaotong University, Xi’an 710004, China; liqiuying@stu.xjtu.edu.cn; 2 Clinical Research Center of Shanxi Province for Dental and Maxillofacial Diseases, College of Stomatology, Xi’an Jiaotong University, Xi’an 710004, China; 3 State Key Laboratory for Manufacturing System Engineering, Xi’an Jiaotong University, Xi’an 710049, China; 4 Department of Physiology and Pathophysiology, School of Basic Medical Sciences, Xi’an Jiaotong University, Xi’an 710061, China; sho_nagi@163.com (C.F.);; 5 Department of Pathology, Xijing Hospital, Fourth Military Medical University, Xi’an 710032, China

**Keywords:** H&E staining, virtual staining, CycleGAN, dual-modal autofluorescence, digital pathology

## Abstract

**Background/Objective:** Hematoxylin and eosin (H&E) staining remains the routine reference in histopathology because it provides essential structural information for tissue evaluation. However, conventional H&E staining involves multiple reagent-based and operator-dependent procedures that may introduce variability in staining appearance. This study aimed to develop and preliminarily evaluate a technical proof-of-concept framework for generating H&E-like images from dual-modal autofluorescence microscopy data using a CycleGAN-based virtual staining approach. **Methods:** Human liver tissue sections were imaged using dual-modal autofluorescence, including DAPI-based nuclear fluorescence and endogenous tissue autofluorescence. A CycleGAN-based virtual staining framework with structural and perceptual constraints was developed to generate H&E-like images, followed by stain normalization to improve color consistency. The generated images were preliminarily evaluated by means of visual comparison, image-feature analysis, feature-space visualization, and nuclear counting. **Results:** The proposed framework generated H&E-like images with encouraging overall visual resemblance to conventional H&E images. Preliminary image-feature analysis suggested partial similarity between virtual and real H&E images, while feature-space visualization indicated that detectable differences remained. Nuclear counting on 168 images showed broadly consistent nuclear distribution between dual-modal autofluorescence and virtual H&E images, with minor discrepancies mainly related to thresholding artifacts. Some fine nuclear and chromatin-level details remained insufficiently reproduced in the current virtual H&E images. **Conclusions:** This study presents preliminary feasibility evidence for a technical proof-of-concept framework that translates dual-modal autofluorescence images into H&E-like images. In this small liver-tissue dataset, the generated images demonstrated encouraging overall H&E-like appearance and approximate nuclear localization. Further studies with larger and more diverse datasets, external validation, expert pathological assessment, diagnostic concordance analysis, and systematic workflow evaluation are warranted to assess robustness, diagnostic relevance, and potential utility in digital pathology workflows.

## 1. Introduction

Histopathological examination serves as a foundation for disease diagnosis, therapeutic planning, and prognostic evaluation [1,2,3]. Among various staining methods, hematoxylin and eosin (H&E) staining has established itself as the routine reference technique in pathology, due to its remarkable capacity to convey comprehensive tissue information, including nuclear details, cytoplasmic morphology, and overall tissue architecture [4,5,6,7]. These features make H&E staining indispensable for visualizing cellular structures and recognizing pathological changes across a wide spectrum of diseases [8,9,10,11]. However, conventional H&E staining involves multiple chemical steps, requires skilled personnel, and is susceptible to variability related to reagents and operational procedures, which may reduce reproducibility and prolong turnaround time [12,13,14]. These limitations have motivated continued efforts to explore complementary digital and computational strategies for histological image generation [15,16,17,18].

In recent years, virtual staining has emerged as a promising digital approach for generating H&E-like images directly from label-free or minimally labeled tissue sections [19,20]. By reducing reliance on conventional chemical staining procedures, virtual staining has attracted increasing interest as a computational approach for generating stain-like histological images and for exploring more standardized image-based pathology workflows [21,22,23]. Nevertheless, many existing virtual staining strategies rely primarily on single-channel autofluorescence signals, which may provide insufficient contrast for simultaneously representing nuclear and cytoplasmic structures. This limitation can affect the morphological fidelity of generated images and may restrict their interpretability.

To address this issue, we developed a proof-of-concept framework for H&E-like virtual staining that uses dual-modal autofluorescence signals—DAPI-based nuclear fluorescence and endogenous tissue autofluorescence—as complementary inputs for CycleGAN-based image translation, followed by stain normalization to improve color consistency. Using a small liver-tissue dataset, we preliminarily evaluated the framework through visual comparison, image-feature analysis, feature-space visualization, and nuclear counting. Further validation in larger and more diverse datasets will help define the robustness, diagnostic relevance, and future role of this strategy in digital pathology workflows.

## 2. Materials and Methods

### 2.1. Ethics Statement and Patient Consent

This study utilized pathological liver tissue sections obtained from five human patients. Written informed consent was obtained from all participants prior to sample collection. Detailed diagnostic classification and systematic histological stratification of the included liver tissue sections were not performed in this preliminary technical proof-of-concept study. The study was approved by the Ethics Committee of Xi’an Jiaotong University (Approval Number: XJTUAE2024-2600).

### 2.2. Image Acquisition and Dataset Preparation

Paraffin-embedded pathological liver tissue sections (2 μm) were dewaxed in xylene (Sinopharm Chemical Reagent Co., Ltd., Shanghai, China), mounted on glass slides, and covered with cover glasses (Thermo Fisher Scientific, Waltham, MA, USA). Nuclei were stained with DAPI (Sigma-Aldrich, St. Louis, MO, USA), and dual-modal autofluorescence (DAF) imaging was performed under a fluorescence microscope (Carl Zeiss AG, Oberkochen, Germany) to acquire both DAPI fluorescence and tissue autofluorescence signals. The excitation wavelengths were 358 nm and 488 nm.

After DAF imaging, the cover glasses were removed by immersing the slides in xylene for approximately 48 h, followed by rinsing with absolute ethanol and 95% ethanol (Sinopharm Chemical Reagent Co., Ltd., Shanghai, China) or 30 cycles each, and a brief wash with deionized water. The same sections were subsequently subjected to conventional H&E staining (Servicebio, Wuhan, China), and whole-slide images (WSIs) were acquired using a whole-slide scanner (3DHISTECH Ltd., Budapest, Hungary). Both DAF and real H&E images from the same sections were used to construct the dataset. After model training, only DAF images were used as input for virtual staining.

The dataset included 5 whole-slide images (WSIs) and 439 regions of interest (ROIs) from 5 patients. A total of 2352 patches extracted from samples of 3 patients were used for model training. For model evaluation, 384 patches were cropped from samples of the remaining 2 patients, including 216 patches for cosine similarity analysis and 168 patches for nuclear counting. No separate validation split was used in this preliminary proof-of-concept study. Train/test separation was performed at the patient level rather than at the slide, ROI, or patch level; therefore, patches from the same patient or WSI did not appear in both the training and test sets. All evaluation patches were derived from the held-out patient-level test set and were cropped without spatial overlap or repetition.

DAF imaging was performed using a 40× objective lens with a numerical aperture of 0.95 (Carl Zeiss AG, Oberkochen, Germany). The emission ranges were 380–399 nm for DAPI fluorescence (exposure time: 1 ms) and 490–507 nm for autofluorescence (exposure time: 50 ms). Each acquired image was larger than 10 GB. For model training, regions of interest (ROIs) were extracted from the WSIs and cropped into 256 × 256 patches using OpenCV (version 4.8.0). During testing, DAF images were similarly divided into patches, processed by the trained network, and then stitched to generate full-slide virtual H&E images.

### 2.3. Virtual Staining Framework

#### 2.3.1. Network Architecture

**Figure 1:**
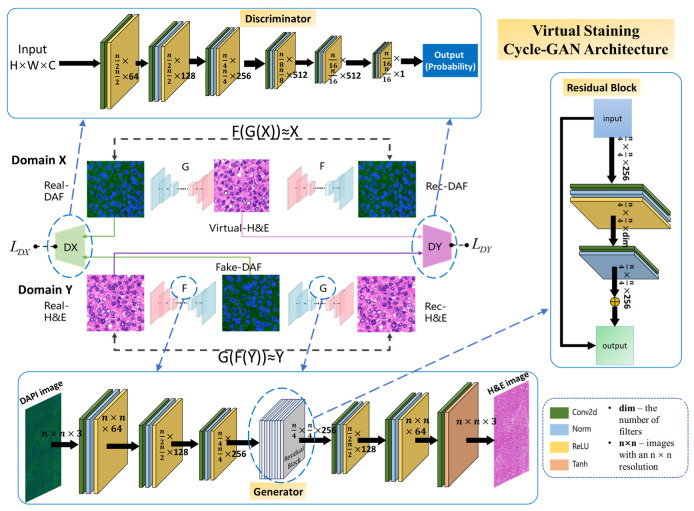
Virtual staining Cycle-GAN architecture. Overview of the virtual staining framework, including the processing workflow, the convolutional architecture of the residual block, the generators (G and F), and the discriminators (D_X_ and D_Y_).

A CycleGAN model comprising two generators (*G* and *F*) and two discriminators ($D_{X}$ and $D_{Y}$) was used for virtual staining. The overall framework and the detailed architectures of the generator and discriminator are shown in Figure 1.

DAF images were input into generator *G* to generate fake H&E images. These generated images were then passed through generator F to reconstruct DAF images, referred to as reconstructed DAF (Rec-DAF) images. Conversely, real H&E images were first transformed by generator *F* into fake DAF images, which were subsequently passed through generator *G* to reconstruct H&E images, referred to as reconstructed H&E (Rec-H&E) images.

Discriminator $D_{X}$ was used to distinguish real DAF images from generated fake DAF images, whereas discriminator $D_{Y}$ was used to distinguish real H&E images from generated fake H&E images. Through adversarial training, the generators learned bidirectional mapping relationships between the DAF and H&E domains.

#### 2.3.2. Loss Functions

To preserve the structural content of DAF images during virtual staining, a cycle-consistency constraint was introduced into the CycleGAN framework. Specifically, a DAF image was first transformed by generator *G* into a virtual H&E image and then mapped back to the DAF domain by generator *F*, yielding a reconstructed DAF (Rec-DAF) image. The loss was calculated between the reconstructed DAF image and the original DAF image, encouraging F(G(x)) to remain close to x. Conversely, a real H&E image was first transformed by generator *F* into a fake DAF image and then mapped back by generator *G* to generate a reconstructed H&E (Rec-H&E) image, encouraging G(F(y)) to remain close to y. These bidirectional mappings enabled generators *G* and *F* to perform style translation while preserving the original image content.

The objective functions for the mapping relationships of generators *G* and *F* are defined as follows:

(1) $$\left\{\begin{array}{l} L_{GAN}(G,D_{Y},X,Y)=E_{y\sim P(y)}[\mathrm{lg}D_{Y}(y)]+E_{x\sim P(x)}\{\mathrm{lg}\{1-D_{Y}[G(x)]\}\}\\ L_{GAN}(G,D_{X},X,Y)=E_{x\sim P(x)}[\mathrm{lg}D_{X}(y)]+E_{y\sim P(y)}\{\mathrm{lg}\{1-D_{X}[G(x)]\}\} \end{array}\right.$$

where x~P(x) and y~P(y) represent the original data distributions of DAF images and H&E images, respectively; $L_{GAN}$ denotes the loss function of the discriminator; and *E* represents the mathematical expectation.

To further improve the quality of virtual staining, Structural Similarity Index (SSIM) loss and Learned Perceptual Image Patch Similarity (LPIPS) loss were incorporated into the original loss function. These additional constraints were introduced to better align the generated images with human visual perception, thereby improving both structural fidelity and perceptual similarity to real H&E images.

SSIM was used to evaluate the similarity of structural information, luminance, and contrast between two images. Compared with conventional metrics such as Mean Squared Error (MSE) and Peak Signal-to-Noise Ratio (PSNR), SSIM is generally considered to be more consistent with human visual assessment. Its mathematical expression is given as follows:

(2) $$SSIM(x,y)=\frac{\left(2\mu_{x}\mu_{y}+C_{1})(2\sigma_{xy}+C_{2}\right)}{\left(\mu_{x}^{2}+\mu_{y}^{2}+C_{1})(\sigma_{x}^{2}+\sigma_{y}^{2}+C_{2}\right)}$$

(3) $$LPIPS(x,y)=\sum_{l}\frac{1}{H_{l}W_{l}}{\sum_{h,w}\left\|w_{l}⊙(\phi_{l}{\left(x\right)}_{h,w}-\phi_{l}{\left(y\right)}_{h,w})\right\|}_{2}^{2}$$

where x and y represent the two images being compared.$\;\phi_{l}$ represents the feature map of the neural network at the *l*-th layer, and *ω*_*l*_ is a weight vector (which can be trainable or fixed) used to adjust the influence of each layer’s feature on the similarity measure. $H_{l}$ and $W_{l}$ represent the height and width of the feature map at the *l*-th layer, respectively. The symbol ⊙ denotes element-wise multiplication, which is used to adjust the weight of the feature differences.

**Figure 2:**
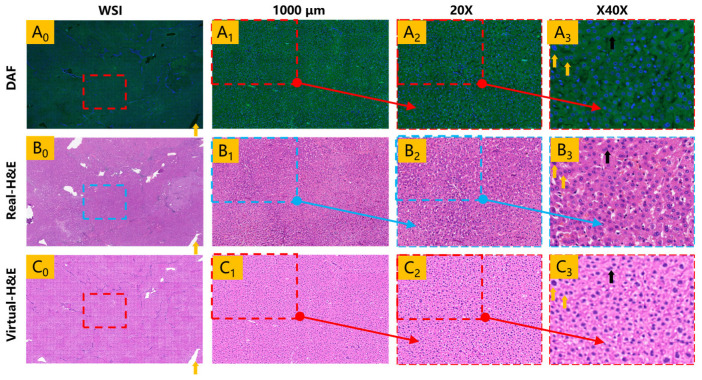
Multi-scale comparison of dual-modal autofluorescence (DAF), real H&E, and virtual H&E images. Rows (A–C) show DAF, real H&E, and virtual H&E images, respectively. Columns (0–3) correspond to the whole-slide image (WSI), a magnified regional view (scale bar = 1000 μm), a 20× view, and a 40× view. Dashed boxes indicate the regions selected for progressive magnification, and the rectangular boxes in the image represent the regions of interest within the current field of view, which are enlarged and displayed with corresponding colors. Virtual H&E images showed an overall H&E-like appearance and approximate nuclear localization across different magnification levels, while differences remained in blank regions, local texture, and fine nuclear details. Yellow arrows indicate cavity-like or blank regions that were more prominent in the real H&E image, whereas black arrows indicate representative nuclear regions in the real and virtual H&E images.

Therefore, the cycle loss terms for the two mapping directions, namely, CycleA_loss (DAF → Virtual-H&E → Rec-DAF) and CylceB_loss (H&E → Fake-DAF → Rec-H&E), are defined as follows:

(4) $$\begin{array}{l} CycleA\_loss=E_{x\sim P(x)}[\lambda_{1}{\left\|F(G(x))-x\right\|}_{1}+\lambda_{2}(1-SSIM(F(G(x)),x))+\lambda_{3}\cdot LPIPS(F(G(x)),x)]\\ CycleB\_loss=E_{y\sim P(y)}[\lambda_{1}{\left\|F(G(y))-y\right\|}_{1}+\lambda_{2}(1-SSIM(F(G(y)),y))+\lambda_{3}\cdot LPIPS(F(G(y)),y)] \end{array}$$

where x~P(x) represents real images from the DAF image domain *x*. G(x) represents the generator *G*, which converts DAF images into Virtual-H&E images. F(G(x)) represents the generator *F*, which converts Virtual-H&E images back into Rec-DAF images. y~P(y) represents real images from the H&R image domain *y*. G(y) represents the generator *F*, which converts H&E images into Fake-DAF images. F(G(x)) represents the generator *F*, which converts Fake-DAF images back into Rec-H&E images. ‖‖_1_ represents L1 loss. λ_1_, λ_2_, and λ_3_ represent weighting factors for the L1, SSIM, and LPIPS loss term, respectively. Figure 2B presents the changes of $CycleA_{\_loss}$ and $CycleB_{\_loss}$ during the training process.

#### 2.3.3. Stain Normalization

This invention introduces a staining normalization technique in the post-processing phase of virtual staining to standardize Virtual-H&E colors, ensuring consistency with reference images. By correcting color deviations, it enhances visual consistency, improving diagnostic accuracy and the reliability of computer-aided systems. This method boosts the readability of Virtual-H&E images, aiding pathologists in efficient interpretation and supporting intelligent pathological diagnosis.

To achieve the normalization of stained images, the first step is to perform stain separation, converting the RGB image obtained after H&E staining into a staining base matrix *W* and staining density matrix *H*. Based on the Beer–Lambert law, which states that stained tissues absorb specific light spectra based on the type and amount of stain, the relative light density matrix when scanning the tissue is represented as follows:

(5) $$V=\log\frac{I_{0}}{I}$$

The staining separation is performed again by utilizing a sparse non-negative matrix factorization on the matrix *V*, in order to obtain the staining base matrix *W* and the staining density matrix *H*, as follows:

(6) V=W·H

And then the stained image after processing is

(7) $$I=I_{0}\exp(-W\cdot H)$$

### 2.4. Training Details

The image size is adjusted to 256 × 256, with a batch size of 1, meaning that 1 image is used for each training step. The model is trained for 200 epochs, with a learning rate of 0.0002 for the first 100 epochs and a learning rate of 0.0001 for the remaining 100 epochs. The Adam optimizer is used with a hyperparameter of 0.5. The experiment is implemented using the Python 3.8 programming language.

### 2.5. Evaluation Metrics

Cosine similarity was calculated only on held-out patient-level test patches, which were not used during model training. The virtual H&E and corresponding real H&E patches were generated from matched regions of the same tissue sections after image alignment and patch extraction. For each paired image, cosine similarity was calculated by flattening the images into pixel vectors and comparing their vector-level similarity. Strict exclusion of background or low-tissue-content patches was not performed before this analysis; therefore, this metric was interpreted as a preliminary global image-feature measure rather than as a local histological or diagnostic similarity metric. Other exploratory evaluations, including perceptual hashing, feature-space visualization, and nuclear counting, were also used as preliminary technical assessments rather than diagnostic validation endpoints.

## 3. Results

### 3.1. Multi-Scale Comparison of Virtual-H&E and Real-H&E Images

Virtual-H&E images showed an overall H&E-like appearance compared with the corresponding real H&E images across different magnification levels. As shown in Figure 2, the generated images reproduced the general color pattern and overall tissue architecture of H&E staining to a certain extent, while differences remained in blank regions, local texture, and fine nuclear details.

At the whole-slide level (A_0_, B_0_, and C_0_), the virtual H&E image showed a general color pattern similar to that of the real H&E image. Nuclei were displayed in blue-purple tones, whereas other tissue components appeared pink, consistent with the main color characteristics of conventional H&E staining. As indicated by the yellow arrows, some blank or cavity-like regions differed among the three image types. Multiple blank areas were visible in the real H&E image, whereas the corresponding regions in the DAF and virtual H&E images appeared less prominent or more uniform, possibly reflecting differences introduced during slide processing, staining, or image generation.

At intermediate magnification (A_1_, B_1_, and C_1_), the virtual H&E image showed comparable overall tissue architecture and color distribution to the real H&E image. The contrast between nuclei and surrounding tissue components was partially preserved, and the general tissue texture distribution observed in the fluorescence image was maintained to a certain extent.

At higher magnification (A_2_, B_2_, C_2_, A_3_, B_3_, and C_3_), the virtual H&E image preserved approximate nuclear locations and overall nuclear density. As indicated by the black arrows, representative nuclear regions were recognizable in the virtual H&E image. However, fine nuclear texture and chromatin-level details were less clearly reproduced compared with real H&E images. Together, these findings indicate that the proposed framework can generate preliminary H&E-like images across multiple magnification levels, while further optimization is needed to improve fine nuclear-detail reconstruction.

### 3.2. Cosine Similarity Analysis as a Preliminary Global Image-Feature Assessment

**Figure 3:**
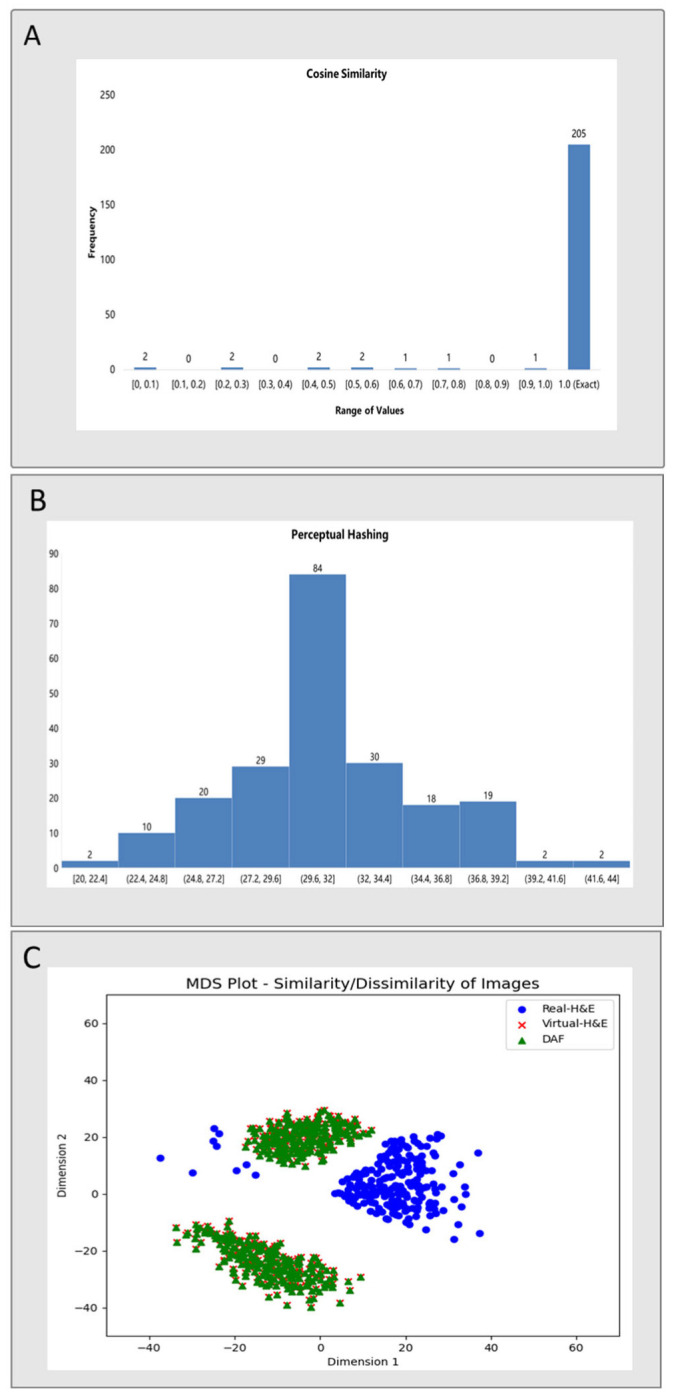
Quantitative analyses of virtual H&E and corresponding real H&E images. (**A**) Cosine similarity between virtual H&E and real H&E images. (**B**) Perceptual hash differences between virtual H&E and real H&E images. (**C**) Multidimensional scaling (MDS) plot showing the similarity and separation among virtual H&E, real H&E, and DAF images.

Cosine similarity was used as a preliminary global image-feature metric to compare virtual H&E and real H&E image patches. A total of 216 held-out test patches were analyzed, and the statistical results are summarized in Table 1. The corresponding frequency distribution is shown in Figure 3A. The mean cosine similarity was 0.9688, with a median of 1.0, and 205 image patches (94.60%) reached a value of 1.0. However, these values should be interpreted cautiously because cosine similarity is a global vector-based metric and may be influenced by uniform background regions and global color or brightness distributions. Therefore, the cosine similarity results were considered only as preliminary global image-feature evidence rather than as evidence of local histological fidelity.

### 3.3. Perceptual Hashing Analysis

Perceptual hashing was used as a supplementary descriptive metric to compare the global visual patterns of virtual H&E and real H&E images. Unlike cosine similarity, which compares flattened pixel vectors, perceptual hashing converts each image into a binary hash representation and measures the Hamming distance between paired images. The distribution of perceptual hash differences is shown in Figure 3B. The mean perceptual hash difference was 31.40, with a median of 32. Most values ranged from 30 to 34, with the first quartile (Q1) at 28 and the third quartile (Q3) at 34.

These results indicate that measurable global visual differences remained between virtual H&E and real H&E images. Because the interpretation of Hamming distance depends on the hash algorithm, hash length, and predefined thresholds, the perceptual hash results were interpreted cautiously as a supplementary visual comparison rather than as definitive evidence of minor or major perceptual difference. Image pairs with larger hash differences generally corresponded to regions with more complex edges, local textures, or tissue structures, suggesting that fine-detail reconstruction remains challenging in the current framework.

### 3.4. Feature-Space Visualization by Multidimensional Scaling

Multidimensional scaling (MDS) was used as an exploratory feature-space visualization method to examine the relationships among DAF, virtual H&E, and real H&E images. MDS preserves relative distances among data points in a low-dimensional space, thereby allowing a visual comparison of feature-distribution patterns across different image types. As shown in Figure 3C, real H&E images formed a relatively compact cluster, whereas DAF images and virtual H&E images were positioned closer to each other in the feature space. The noticeable separation between real H&E and the other two groups suggests that the generated virtual H&E images did not fully overlap with the real H&E feature distribution. These findings indicate that the current virtual staining framework achieved partial domain translation from DAF to H&E-like images, while the generated images still retained certain feature characteristics of the DAF input. Therefore, the MDS results were interpreted as exploratory evidence of incomplete feature-space transformation rather than as direct evidence of equivalence between virtual H&E and real H&E images.

### 3.5. Preliminary Nuclear Localization Assessment in Virtual H&E Images

**Figure 4:**
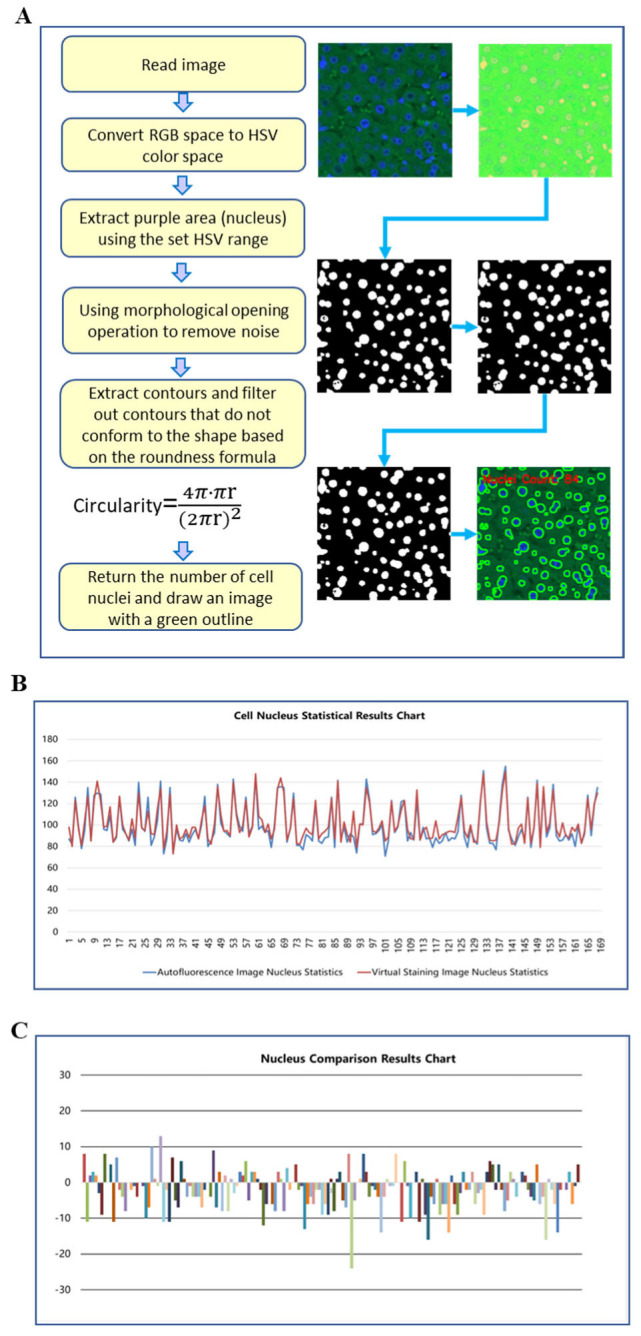

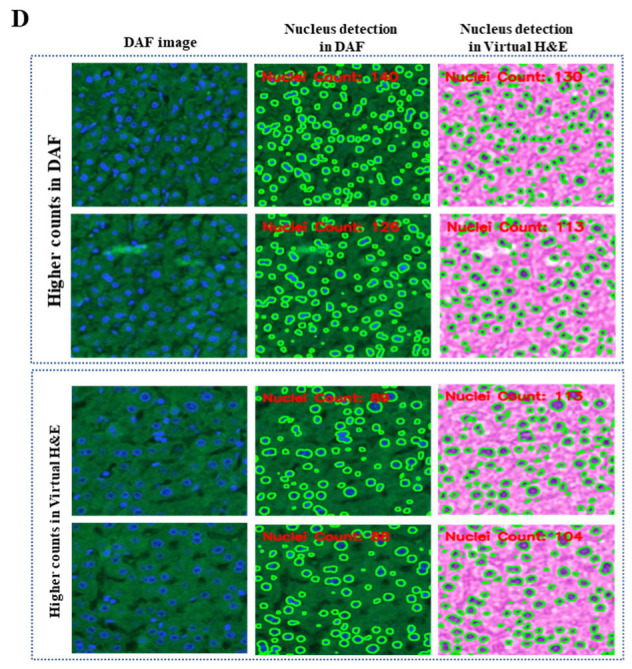
Preliminary nuclear localization assessment in virtual H&E images. (**A**) Workflow for HSV-thresholding-based nuclear counting. (**B**) Comparison of nuclear counts between virtual H&E images and corresponding DAF images. (**C**) Differences in nuclear counts between virtual H&E and DAF images. (**D**) Representative examples showing discrepancies in nuclear counts between DAF and virtual H&E images. The three columns show the original DAF image, the nucleus detection result in the DAF image, and the nucleus detection result in the corresponding virtual H&E image, respectively. Green contours indicate detected nuclear regions, and the count label shows the total number of detected nuclei. The upper panel presents examples with higher nuclear counts in DAF images, whereas the lower panel presents examples with higher nuclear counts in virtual H&E images. Arrows indicate representative regions contributing to the discrepancy.

To preliminarily evaluate nuclear localization in virtual H&E images, 168 held-out test images were selected, and HSV-thresholding-based nuclear counts were compared with those in the corresponding DAF images. The workflow used for nuclear quantification is shown in Figure 4A. Because DAPI directly labels nuclei, DAF images were used as a technical reference for nuclear localization rather than as a clinically relevant comparator to real H&E images. As shown in Figure 4B, the nuclear count curves of virtual H&E and DAF images showed broadly similar trends, suggesting that the virtual H&E images preserved approximate nuclear localization relative to DAF images. However, discrepancies were observed at several data points, including x-coordinates 7 and 64. The differences in nuclear counts are further summarized in Figure 4C, where positive values indicate higher counts in DAF images and negative values indicate higher counts in virtual H&E images. In this analysis, virtual H&E images showed a tendency toward slightly higher nuclear counts.

Representative cases with the largest absolute differences are presented in Figure 4D. Higher counts in DAF images were mainly caused by erroneous identification of black holes as nuclei or missed detection of stained nuclei in virtual H&E images. In contrast, higher counts in virtual H&E images were mainly related to enhanced tissue textures overlapping with the HSV threshold, which led to misclassification of non-nuclear regions as nuclei.

Taken together, these results suggest approximate preservation of nuclear localization in virtual H&E images relative to DAF images. However, because this analysis was based on a simple HSV-thresholding method and did not include real H&E-based nuclear detection or expert-validated nuclear annotations, it should be interpreted only as a preliminary technical assessment rather than as definitive evidence of nuclear fidelity.

## 4. Discussion

In this study, we developed a virtual staining framework that integrates dual-modal autofluorescence imaging with stain normalization to generate H&E-like images from liver tissue sections without conventional chemical staining. By combining DAPI-based nuclear fluorescence with endogenous tissue autofluorescence, the framework incorporates complementary information on nuclear localization and broader tissue structure into the image-generation process. The generated images reproduced the overall H&E-like color pattern, tissue architecture, and approximate nuclear localization, supporting the technical feasibility of this strategy for virtual H&E image generation. These findings provide a foundation for further optimization and validation in larger and more diverse pathological datasets.

An important feature of the present framework is the incorporation of stain normalization. In digital pathology, color inconsistency remains a practical challenge for both human interpretation and image-based computational analysis [3]. By integrating stain normalization into the virtual staining pipeline, we aimed to improve the consistency of color appearance in the generated images and reduce variability related to acquisition conditions. This extends the framework beyond image generation alone by also addressing the stability of the resulting color representation [8,9,10]. Such an approach may support more consistent virtual H&E image presentation across broader datasets and imaging settings, while its performance under different sample types, platforms, and pathological conditions should be further evaluated.

The dual-modal design was intended to integrate complementary information from DAPI fluorescence and endogenous tissue autofluorescence. DAPI fluorescence provides explicit nuclear information, whereas endogenous autofluorescence contributes broader tissue texture and structural context. The combination of these two signals may therefore support a more balanced representation of nuclear and non-nuclear tissue components in virtual H&E images, which is consistent with previous virtual staining studies suggesting that richer optical input or optimized model design can improve histological image reconstruction [3,10,19]. In our multi-scale comparisons, the generated images reproduced the general color pattern and overall tissue architecture of conventional H&E staining to a certain extent. Fine nuclear texture and chromatin-level details remained less clearly reconstructed, indicating an important direction for further model optimization. Because single-modal comparison groups were not included in the present study, the relative contribution of each modality will be further evaluated through formal ablation analysis in future work.

Our findings complement several representative studies in the literature. Rivenson et al. highlighted the feasibility of transforming tissue autofluorescence images into virtually stained histology images using deep learning, whereas Salido et al. compared multiple deep learning models for digital H&E staining from unpaired label-free multispectral microscopy images and showed that model selection can significantly affect reconstruction quality [8,10]. Koivukoski et al. further demonstrated the feasibility of generating virtual H&E whole-slide images from unstained tissue imaging [19]. Building on these studies, the present work explores the integration of dual-modal autofluorescence input and stain normalization within a CycleGAN-based framework for H&E-like image generation in liver tissue. This design provides a complementary proof-of-concept strategy that incorporates both nuclear fluorescence and broader tissue-structural information while also addressing color consistency in the generated images.

From an application perspective, the proposed framework may provide a complementary approach for generating H&E-like images in settings where conventional staining workflows are time-consuming or operationally demanding. The encouraging visual resemblance observed in this study supports its potential value for further development. To establish clinical relevance, future validation should include blinded expert assessment, interobserver agreement analysis, and diagnostic concordance testing using spatially matched real and virtual H&E images [3].

Several limitations should be considered when interpreting the present findings. The study was based on a relatively small liver-tissue dataset with limited histological heterogeneity, and detailed diagnostic classification and systematic histological stratification were not performed. All samples were obtained and imaged at a single institution using one imaging platform and a consistent acquisition workflow. This single-institution setting may limit the generalizability of the present findings, as variations in tissue preparation, microscope configuration, illumination, exposure settings, image resolution, and preprocessing procedures could affect model performance across institutions and platforms. Cross-institutional validation using independently acquired datasets and different imaging systems is therefore required to assess the robustness and transportability of the proposed framework.

Future studies should include larger and more histologically diverse datasets, together with cross-institutional validation using independent imaging systems. Formal ablation analysis and pathologist-based assessment will also be needed to clarify the contribution of each input modality and evaluate histological quality and diagnostic interpretability [3,10,19]. The framework may also be extended to multimodal or multiplex imaging settings that integrate structural and molecular information, helping to define the robustness, generalizability, and potential role of dual-modal virtual staining in digital pathology workflows.

## Figures and Tables

**Table 1:** Statistical Results of Cosine Similarity.

Range of Values	Frequency	Proportion
[0, 0.1)	2	0.92%
[0.1, 0.2)	0	0.00%
[0.2, 0.3)	2	0.92%
[0.3, 0.4)	0	0.00%
[0.4, 0.5)	2	0.92%
[0.5, 0.6)	2	0.92%
[0.6, 0.7)	1	0.46%
[0.7, 0.8)	1	0.46%
[0.8, 0.9)	0	0.00%
[0.9, 1.0)	1	0.46%
1.0 (Exact)	205	94.90%

## Data Availability

The original contributions presented in this study are included in the article. Further inquiries can be directed to the corresponding author.
